# The Influence of Acid Whey on the Lipid Composition and Oxidative Stability of Organic Uncured Fermented Bacon after Production and during Chilling Storage

**DOI:** 10.3390/antiox10111711

**Published:** 2021-10-27

**Authors:** Anna Okoń, Piotr Szymański, Dorota Zielińska, Aleksandra Szydłowska, Urszula Siekierko, Danuta Kołożyn-Krajewska, Zbigniew J. Dolatowski

**Affiliations:** 1 Department of Meat and Fat Technology, Prof. Wacław Dąbrowski Institute of Agricultural and Food Biotechnology-State Research Institute, 36 Rakowiecka St, 02-532 Warsaw, Poland; piotr.szymanski@ibprs.pl (P.S.); urszula.siekierko@ibprs.pl (U.S.); zbigniew.dolatowski@ibprs.pl (Z.J.D.); 2 Institute of Human Nutrition Sciences, Warsaw University of Life Sciences (WULS), Nowoursynowska St. 159C, 02-776 Warszawa, Poland; dorota_zielinska@sggw.edu.pl (D.Z.); aleksandra_szydlowska@sggw.edu.pl (A.S.); danuta_kolozyn_krajewska@sggw.edu.pl (D.K.-K.)

**Keywords:** uncured fermented bacon, acid whey, oxidative stability, fatty acid profile

## Abstract

The aim of this research was to evaluate the effect of acid whey on changes in the fatty acid profile, oxidative stability, physico-chemical parameters, and microbiological and sensory quality of traditional organic uncured fermented Polish bacon after production and during chilling storage. Three different treatments of fermented bacon were produced: C—control bacon with a nitrite curing mixture; T—bacon with a nitrate curing mixture; and AW—bacon with acid whey and NaCl. The acid whey used in the production of uncured fermented pork bacon positively changed the sensorial characteristics, directly after the ripening process, and had a positive effect in terms of a decrease in the pH of the product. All of the fermented bacon treatments in general were of good microbiological quality. A higher lactic acid bacteria (LAB) level was observed in the AW treatment after the fermentation process, and the bacteria number did not change during storage, whereas in the C and T treatments, the LAB level increased during storage (*p* < 0.05). The application of acid whey did not limit the formation of secondary oxidation products (TBARS) during bacon ripening (1.68 mg MDA kg^−1^), but had a reduced value during storage time (0.73 mg MDA kg^−1^). The highest polyunsaturated fatty acid (PUFA) levels, after ripening and after four weeks of refrigerated storage, were found in the C treatment. In the AW treatment, it was found that the PUFA level increased; likewise, the content of n-3 and n-6 fatty acids increased, while saturated fatty acids (SFAs) decreased during storage (*p* < 0.05). The opposite tendency was observed in the C treatment. After four weeks of storage, the PUFA/SFA ratio was the lowest in the nitrate treatment, and higher values of the PUFA/SFA ratio were obtained in the acid whey and nitrite treatment (*p* < 0.05).

## 1. Introduction

Traditional organic food products have been increasingly regaining consumer interest worldwide. This is thanks to their unique sensory properties, high quality, and natural composition. They are closely connected to the culture, heritage, and local identity of a given population, and they also have a strong symbolic value and contribute to the sustainability and development of rural areas [1,2]. An important principle of organic product practice is that producers use very few food additives during processing. The difficulty in the production of organic meat products is the elimination of some substances, such as nitrite and nitrate [3,4]. Nitrite and/or nitrate are widely used as preservatives in meat production. Nitrate serves as a precursor to nitrite in raw cured fermented meat products [3,5]. The use of nitrite exerts a significant antimicrobial effect related to the inhibition of the growth of several pathogens such as *Listeria monocytogenes*, *Clostridium botulinum*, and *Salmonella* [3,6,7]. Nitrite is responsible for the characteristic reddish-pink color and the flavor of cured meats. Finally, nitrite can prevent deterioration by inhibiting lipid peroxidation [2,3]. Regardless of the benefits of using sodium nitrite, it has the potential to participate in the formation of cancerogenic N-nitroso compounds, which can be formed in the food matrix as well as in the human body [3]. Meat products without or with reduced added nitrite/nitrate are characterized by high consumer acceptance [8,9,10,11]. Thus, the food industry has tried to replace synthesized nitrite with the use of vegetable extracts [10,12,13,14] and/or natural antimicrobials or the direct addition of bioprotective lactic acid bacteria cultures [6,15,16,17] and the strict control of the manufacturing process [18,19].

Numerous promising studies have been carried out to replace nitrite in organic meat products with acid, whey but none of them have discussed meat products with a high fat content such as pork bacon [9,20,21,22]. Many authors have indicated that there are specific issues related to the flavor, safety, and quality of processed meat products with a high fat content with no or low nitrite concentrations [7,19,23].

The oxidation of lipids generates the production of toxic, cancerogenic substances that are harmful to human health [24]. The oxidative stability of meat products depends on the equilibrium of anti- and pro-oxidants and the composition of easily oxidative substances, i.e., polyunsaturated fatty acids (PUFAs), cholesterol, proteins, and heme pigments [25,26]. The oxidation and rancidity of meat are mainly affected by the participation of polyunsaturated fatty acids, as well as the level of cholesterol, mainly including low-density lipoprotein (LDL). Acid whey (AW) may be an important element in the oxidation process of products. Depending on the production technology of cottage (white fresh) cheeses, two types of whey are produced: Rennet (“sweet”) whey, obtained during the manufacturing of maturing cheeses, and acidic (“acid”) whey, coming from cottage cheese production. Acid whey (pH 3.8–4.6) is characterized by a higher content of lactic acid (up to 0.7%) and a lower content of proteins compared to rennet whey [27]. The whey consists of proteins, peptides, lactose, calcium and phosphorus compounds, organic acids and vitamins from the B group, and many antioxidative compounds (glutathione, lactoferrin, etc.). Organic acid whey is the source of lactic acid bacteria, mainly *Lactobacillus*, which is the predominant microbiota [27,28]. Furthermore, some researchers have reported that some species of *Lactobacillus* have considerable lipolytic activity and lactic acid bacteria lipases are involved in the lipid metabolism in fermented meat products [29,30].

The objective of the present study was to evaluate the effect of acid whey on changes in the fatty acid profile, oxidative stability, physico-chemical parameters, and microbiological and sensory quality of traditional organic uncured fermented Polish pork bacon after production and during chilling storage.

## 2. Materials and Methods

### 2.1. Materials

#### 2.1.1. Preparation of Acid Whey

The acid whey was obtained from organic cottage cheese production in a dairy processing plant located in Bydgoszcz, Poland. The mean values of the pH and color parameters of the acid whey were, respectively, 4.60 and L* = 33.63, a* = −1.60, and b* = 0.62. The lactic acid bacteria (LAB) concentration in the acid whey was approximately 8.0 log CFU/mL. The count of *Enterobacteriaceae* was 3.48 log CFU/mL and the count of yeast and molds was 2.68 log CFU/mL. In the acid whey, no *Salmonella* sp., *Staphylococcus aureus*, or *Listeria monocytogenes* was confirmed (data are not presented in the table).

#### 2.1.2. Fermented Bacon Production

The raw meat material for the manufacture of the fermented products was pork belly with four ribs cut off. The rib bones was removed from the meat material. The flank muscle and the teat line were removed from the pork belly. The width of the bellies was from 20 to 24 cm and the length from 40 to 44 cm. The porcine cuts were obtained from nine chilled carcasses of Polish Large White adult pigs (bars) weighing between 120 and 130 kg. The weight of the bellies ranged from 1300 to 1600 g, and the meat was free from quality defects. The pigs originated from organic breeding from one litter; they were kept under the same conditions for eight months. The bellies were randomly divided into three experimental batches with three bellies each. Three different treatments for the fermented bacon were produced: C—control bacon with a nitrite curing mixture (99.5% NaCl, 0.5% NaNO2); T—bacon with a nitrate curing mixture (99.5% NaCl, 0.5% NaNO3); and AW—bacon with acid whey and NaCl. At first, in the AW treatment, cold acid whey was added (1% in relation to the meat) to the belly and marinated in plastic containers for 24 h at 2–4 °C. Then, the C treatment was cured using a nitrite curing mixture (1.5% in ratio to the meat), the T treatment was cured using a nitrate curing mixture (1.5% in relation to the meat), and the AW treatment was salted (1.5% in relation to the meat) and stored in separate plastic containers at 2–4 °C for 24 h. Then, the pork belly was left in the fermentation (ripening) chamber at a temperature of 16 °C and a relative air humidity of 70%–75% for 21 days. After five days, the pork belly was smoked with cold smoke at a temperature of 30–35 °C for 40 min. The smoking process was carried out in a traditional smokehouse, with a brick chamber with dimensions of 100 × 100 × 250 cm lined with a clinker inside. At a distance of 100 cm from the chamber, there was a hearth with air supply control. The smoke from the incandescence beech wood billet (without bark) was delivered through a suitable channel from the hearth to the bottom of the chamber. The smoking process was carried out in an open circuit. All of the pork bellies were suspended in the chamber at a height of 100 cm from the bottom of the chamber. After processing, each fermented pork bacon (finished product) was cut into two portions, individually vacuum-packed, and stored at a temperature of 4–6 °C. The mean value of the fat content of the cuts of finished product was 55.2 ± 7.2%. The products were tested after production (time 0) and after four weeks of chilling storage. The entire experiment was repeated three times in the meat processing plant in Bydgoszcz, Poland.

### 2.2. Methods

#### 2.2.1. Microbiological Analyses

The analyses were carried out using the spread plate technique on an appropriate culture medium for a tested group of microorganisms: to determine the TVC (total viable count; nutrient agar—LabM, Heywood, UK; incubation parameters, 37 °C per 48 h in accordance with the ISO 4833-2 was used) [31], LAB (lactic acid bacteria; MRS agar—de Man Rogosa Sharpe agar, Merck, Germany; incubation parameters, 37 °C per 48 h according to ISO 4833-1) [32], Y&M (the number of yeasts and molds; Sabouraud dextrose with Chloramphenicol Lab Agar, Biomaxima, Lublin, Poland; incubation parameters, 25 °C per five days in accordance with ISO 21527-1 and ISO 21527-2) [33,34], ENT (microorganism from the Enterobacteriaceae family; Mac Conkey agar, Merck, Germany; incubation parameters, 30 °C per 24 h according to ISO 21528-2) [35], STA (*Staphylococcus aureus* count; Baird-Parker agar supplemented with egg yolk tellurite, Merck, Germany; incubation parameters, 37 °C per 24–48 h), LIST (*Listeria monocytogenes* presence in 25 g of the sample; ALOA agar—Listeria agar according to Ottaviani and Agosti, Bio-Rad, USA, and PALCAM agar—polymyxin acriflavine lithium chloride ceftazidime esculin mannitol, LabM, UK; incubation parameters, 37 °C per 48 h according to ISO 11290-1) [36]. The obtained results are given in colony forming units per gram of the product (CFU g^−1^).

#### 2.2.2. Determination of the pH Value

The pH value was measured in a mixture obtained by homogenization (14.000 RPM) with 10 g of the minced sample and 50 mL of distilled water for 1 min using a 800 W blender (MSM 66120, BSH Hausgeräte GmbH, Munich, Germany). A digital pH meter (Delta 350, Mettler Toledo, Schwerzenbach, Switzerland) equipped with an In Lab Cool electrode (Mettler Toledo, Greifensee, Switzerland) was used. The pH-meter was standardized with buffer solutions at pH 2.0, 4.0, 7.0, and 10.0. Measurements were performed at 20 °C.

#### 2.2.3. Oxidative Reduction Potential (ORP) Measurement

The ORP value was measured on a 10 g homogenized sample (14.000 RPM) with 50 mL of distilled water for 1 min using an 800 W blender (MSM 66120, BSH Hausgeräte GmbH, Munich, Germany). The redox potential was measured using a digital pH meter (Delta 350, Mettler Toledo, Schwerzenbach, Switzerland) equipped with an In Lab Redox Pro electrode (Mettler Toledo, Greifensee, Switzerland).

The obtained results were calculated into the value of ORP in relation to the standard hydrogen electrode EH(mv). The ORP value of the reference electrode at a temperature of 20 °C Eref = 207 mV was summed up with the value obtained with the equipment. Measurements were performed at 20 °C.

#### 2.2.4. TBARS Value

The index of fat oxidation TBARS (TBARS assay) was determined, measuring the value of the absorbance of the solution of the meat product and 2-thiobarbituric acid described by Pikul, Leszczynski, and Kummerow [26]. The intensity of color, resulting from the reaction of malondialdehyde (MDA) and 2-thiobarbituric acid, was measured using a U-2900 spectrophotometer (Hitachi, Tokyo, Japan) at a wavelength of 532 nm. The value of the TBARS index was expressed in milligrams of malondialdehyde per kilogram of meat product and it was calculated using the following equation:

TBARS (mg MDA kg^−1^ sample) = 5.5 × absorbance

#### 2.2.5. Fatty Acid Profile

The determination of fatty acid composition was conducted by the GC method (HP 6890 II with a flame-ionization detector, Hewlett-Packard, USA) according to PN-EN ISO 12966-1:2015-01 [37]. For the separation of esters, a high polar capillary BPX 70 column (60 m × 0.25 mm, 25 µm) was used. The injector temperature was 240 °C with a split ratio set to 100:1 and the FID temperature was 250 °C. The oven temperature was ramped from 130 °C (1 min) to 210 °C (7 min) at a rate of 1.5 °C min^−^^1^. Helium was used as a carrier gas with a constant pressure of 40 psi at a flow rate of 0.3 mL min^−^^1^ and an injection volume of 1 µL. GC-FID was used to determine the relative percent areas of the FAME components present in the analyzed samples as the reference method. The total analysis time was 61 min. The results of the analysis were automatically calculated according to the principle of internal normalization by the ChemStation software version A 03.34^®^ 1989–1994. Peaks of fatty acid methyl esters resolved by GC were identified by comparison to standard FAME mixtures of known composition.

#### 2.2.6. Sensory Analysis

The sensory quantitative descriptive profile (QDP) method was used (ISO 13299:2016-05) [38]. The descriptors were selected and defined during a panel discussion and then verified in a preliminary session. Seventeen sensory attributes were measured to quantify the quality of the tested products (Table 1). The task of the assessment team was to determine the intensity of the chosen quality features and to put the assessment results on a proper graphic scale (0–10 c.u.).

Fermented pork bacon samples were sliced into approximately equal size and weight (10 g) and placed in odorless, disposable, plastic boxes covered with lids. All of the samples were separately coded for assessment with three digit codes and were passed in random order to avoid the carry-over effect (i.e., the impact of a previous sample on a subsequent one). The test samples were kept in boxes in a room at 22 °C for 30 min before analysis. The assessments were performed by a nine-person trained team. The sensory quality evaluation of the experimental bacon was performed after production (time 0).

### 2.3. Statistical Analysis

All of the experiments were performed in triplicates in independent trials (replicates; *n* = 3) at different times under industrial conditions, and a completely randomized design was used. The results (physicochemical and microbiological analyses) obtained in the research were statistically analyzed using a two-way ANOVA. The model included the treatments effect (nitrite, nitrate, and acid whey) of the time of storage (0 and 4 weeks) of the samples and their interaction (treatment x time of storage). Sensory analysis data was analyzed by one-way ANOVA (between treatments) after production (time 0). The means and standard deviations were calculated. The Fisher post-hoc test was used to determine the significance of the mean values for multiple comparisons (*p* < 0.05). The Statistica package, version 13 (StatSoftPolska Sp. z o.o, Cracov, Poland), was used for the calculations.

## 3. Results and Discussion

### 3.1. Microbiological Assessment

The microbiological assessment of fermented pork bacon after maturing and storage is shown in Table 2. The treatment and time of storage affected (*p* < 0.001) the TVC, LAB, and ENT levels. An interaction between the treatment and time of storage for the TVC, LAB, and ENT levels was also found (Table 2). The pork bacon samples were of good microbiological quality. A similar TVC level ranging between 6.23 and 6.48 log CFU/g was observed, and the level significantly increased after four weeks of storage in the AW and T treatments. In the C treatment, no increase in the TVC level was shown after storage (*p* > 0.05). This is probably the effect of the addition of nitrite in the C treatment, which is known to have a bacteriostatic effect with other substances, mainly with sodium chloride added to meat [4,39]. The treatment was different in the LAB count. A significantly higher LAB level was observed in the AW treatment after the production process, and the bacteria number did not change during storage, whereas in the C and T treatment, the LAB level increased during storage (*p* < 0.05). The high number of lactic acid bacteria in the AW treatment after production was associated with the addition of acid whey to meat, which contained a high level of LAB (10^8^ CFU/mL). In other studies, it was also demonstrated that the traditionally obtained acid whey is characterized by an LAB level ranging from 4 to 5 log CFU/mL up to approximately 8 log CFU/mL; however, the LAB count can differ depending on the source of the samples [29,40]. The microbiological quality of acid whey is of great importance in meat production, as this is a natural source of LAB. No increase in the number of lactic acid bacteria observed in the AW treatment during storage indicates that the conditions in such a meat environment (high fat content and low water activity) were not favorable for the growth of LAB from acid whey.

A significant influence of treatment (*p* < 0.05) and time of storage (*p* < 0.001) on the Y&M level was observed. An interaction between the treatment and time of storage was found for the Y&M level (Table 2). Yeast and molds were at a rather high level in all of the treatments and ranged from 5.70 to 6.58 log CFU/g at the beginning, slightly decreasing to 5.30–5.90 log CFU/g after storage. All of the pork bacon treatments were free of selected pathogens and hygienic status marker bacteria, except for the AW sample at four weeks of storage, where a small number of Enterobacteriaceae family microorganisms were detected.

### 3.2. pH and ORP

The treatment and time of storage affected (*p* < 0.001) the pH value. An interaction between the treatment and time of storage for pH also was found (Table 3). The value of the acidity (pH) of the fermented pork bacon (Table 3) directly after fermentation (0) and after four weeks of refrigerated storage showed the lowest pH value (*p* < 0.05) in the AW treatment with acid whey (5.78 and 5.67, respectively), whereas the highest one was found in the T treatment with sodium nitrate (6.06 and 6.00, respectively). After four weeks of refrigerated storage, a lowering in the pH value (*p* < 0.05) was observed in the AW treatment by 0.11 units (Table 3). The pH value of the meat and meat products is a sign of the changes occurring as a result of biochemical transformations and the increase in microorganisms [41,42]. The higher acidity of the AW treatment was most likely the result of the fermentation caused by lactic acid bacteria derived from acid whey and the accumulation of organic acids, including lactic acid [42]. The highest number of lactic acid bacteria in the AW treatment was demonstrated after production (Table 2); this relationship was not shown after storage. However, it is known that different strains of lactic acid bacteria may have different rates of fermentation and acid production [1,43]. Another factor that could affect the pH value of the AW treatment was the acidity of the acid whey used (pH = 4.60). The higher acidity of raw fermented meat products with the use of acid whey was also observed by other authors in their studies [20,21]. The differences in the acidity of the C and T treatments may have resulted from differences in the metabolism of environmental bacteria, including the LABs in connection with the addition of nitrite or nitrate to meat. Some of the Lactobacillus bacteria strains may reduce nitrate to ammonia in a process called fermentative nitrate reduction or ammonification [5]. The accumulation of certain amounts of ammonia in the meat could have resulted in a higher pH in the T treatment.

A significant (*p* < 0.001) influence of treatment and time of storage on the redox potential value was observed. In addition, an interaction between the treatment and time of storage for redox potential was found (Table 3). The value of the oxidation-reduction potential (ORP) of the fermented pork bacon with the use of acid whey (AW) revealed a significantly (*p* < 0.05) higher level (between 75 and 80 mV) compared to the remaining treatments, examined directly after the ripening process. The lowest ORP value (246.5 mV) was found in the C treatment. The lower value of the redox potential in the C treatment could have been affected by the antioxidative effect of the added nitrite [44]. After four weeks of storage, a significant (*p* < 0.05) lowering of the oxidation-reduction potential only in the treatment with acid whey was found, as well as a significant (*p* < 0.05) increase in the remaining treatments (Table 3).

Lactic acid bacteria applied with the acid whey to the surface of raw belly found an unfavorable environment. LAB belongs to organisms that are relatively anaerobic but under unfavorable conditions, they might use oxygen as a final electron acceptor. The oxygen might have had a negative effect on the lactic acid bacteria cells. One of the products occurring due to oxygen reduction by bacteria within the respiratory chain strongly oxidizes hydrogen peroxide (H_2_O_2_) [45,46,47]. Some amount of H_2_O_2_ produced by the selected bacteria in the raw belly could have had an effect on the higher redox potential value in the AW treatment after the ripening process compared to the other variants. It is known that some LAB respond to oxygen stress with increased anti-oxidative enzyme synthesis, such as pseudocatalse containing manganese or glutathione peroxidase [45,48]. The anti-oxidative enzyme synthesis by LAB from acid whey could have had an impact on the decrease in the redox potential value in the AW treatment after storage.

### 3.3. TBARS Index

The treatment and time of storage affected (*p* < 0.001) the TBARS value. An interaction between the treatment and time of storage for TBARS also was found (Table 4). In the present study, it was found that the TBARS values were significantly affected (*p* < 0.05) by the storage time and the use of additives (nitrite, nitrate, and acid whey) (Table 4). The highest TBARS value was found in the AW treatment with acid whey (1.68 mg MDA kg^−1^) (*p* < 0.05). Significantly lower values were found in the T treatment cured with sodium nitrate (0.71 MDA kg^−1^) and in the C treatment cured with sodium nitrite (0.96 MDA kg^−1^) directly after the ripening process (*p* < 0.05). After four weeks of storage, a lower TBARS value by 0.95 MDA kg^−1^ in the AW treatment and by 0.26 MDA kg^−1^ in the C treatment was observed (*p* < 0.05). Meanwhile, after four weeks of storage, in the T treatment, a significant increase (*p* < 0.05) by 0.91 MDA kg^−1^ in the value of the TBARS index was observed and recorded in comparison to the value measured directly after ripening. After four weeks of storage, the lowest TBARS values were found in the AW treatment with acid whey and in the C treatment cured sodium nitrite. A significantly higher TBARS value was found in the T treatment, cured with sodium nitrate (*p* < 0.05) (Table 4).

A high oxidation-reduction potential favors oxidation reactions, which result in peroxides, hydroperoxides, and secondary fat oxidation products [49]. This correlation can be observed especially in bacon with acid whey after production, where the redox potential and TBARS index were the highest. The obtained results indicate that acid whey does not limit fat oxidation during the production process (fermentation) of raw bacon, but then, during cold storage, the inhibition of oxidative processes and a lowering of the TBARS value in the examined bacon were observed. Libera et al. [50] suggested that the high TBARS index in raw-ripening necks directly after production may be a cause of the reaction of the compounds and amino acids, carbohydrates, nitrates, and nitrites. Karwowska et al. [22] observed a high TBARS index in scalded sausages with the addition of acid whey, whereas the application of mustard (*Sinapis alba* L.) and acid whey in the sausages considerably limited the level of the examined index.

Other authors, when examining fermented meat products with the application of acid whey, have also confirmed the higher values of the TBARS index directly after the ripening process (up to ca. 2 mg MDA kg^−1^) [20,21]. The controlled oxidative changes in raw-ripened products may positively affect the generation of appropriate flavoring, approved by the consumer. The highest value of TBARS after four weeks of storage of the treatment with sodium nitrate may be the reason for unfavorable changes, induced by the product’s microbiota [51].

### 3.4. Fatty Acid Profile

The fatty acid composition of the fermented pork bacon after maturing and storage is shown in Table 5. A sample chromatogram of the fatty acids of the fermented pork bacon with the use of acid whey (AW) after four weeks of storage is shown in the Supplementary Figure S1. The treatment and time of storage affected (*p* < 0.001) the total saturated fatty acid (SFA), monounsaturated fatty acid (MUFA), and PUFA proportions of the fermented pork bacon. An interaction between the treatment and time of storage for SFA, MUFA, and PUFA also was found (Table 5).

The results indicate that the treatment significantly affected the fatty acid profile of the fermented pork bacon (*p* < 0.05). The analysis of the fatty acid profile in the C treatment after fermentation (time 0) revealed the significantly (*p* < 0.05) highest PUFA content (13.75%) and lowest SFA content (34.77%). The opposite tendency was found in the T and AW treatments, where the lowest PUFA content (respectively, 9.75% and 9.55%) and the highest SFA content (respectively, 39.02% and 39.00%) were observed. The C treatment was characterized by a higher content of n-3 and n-6 fatty acids than the other treatments (*p* < 0.05). The monounsaturated fatty acid (MUFA) content was also higher in the nitrite treatment (C) than in the acid whey treatment (AW) after production (*p* < 0.05) (Table 5).

Significantly higher SFA and the lowest PUFA content after production in the acid whey (AW) and nitrate (T) treatments were mainly caused by oxidative changes, because polyunsaturated fatty acids are oxidized faster [49]. One of the main factors influencing fat oxidation in meat and its products is the fat content and the composition of fatty acids. Due to the high content of fat, pork belly is a raw material that is extremely susceptible to oxidative changes, which affect its nutritional value and sensory quality. The composition of fatty acids changes during processing and refrigerated storage, affecting the sensory evaluation and nutritional value of food. Fat oxidation reactions can directly affect the composition of fatty acids and unsaturated fatty acids, which are the most susceptible to oxidative changes [25,49]. Coutron-Gambotti et al. [52] found that during the processing of fermented hams, oxidation of fat leads to a reduction in the amount of long-chain polyunsaturated fatty acids. In our study, the lower percentage of PUFA and the highest levels of TBARS and ORP observed in the acid whey treatment immediately after production suggest that acid whey does not sufficiently protect lipids from oxidation during fermentation as it does in the sodium nitrite treatment. The fatty acid profile of the fermented pork bacon after production indicates that better lipid protection during fermentation occurred in the C treatment, in which highly antioxidant sodium nitrite was used [53]. This is consistent with the literature data [22,54] concerning organic pork sausages with the addition of acid whey. These studies showed a lower content of PUFA after production in sausages with acid whey compared to the cured control samples. These results were confirmed by the better antioxidant properties of nitrite and the higher oxidative stability of the cured sausages.

On the contrary, some studies have shown a positive effect of the use of acid whey on the fatty acid profile in beef sausages [20] and pork loins [11] after production. These products are characterized by a higher PUFA content compared to the cured control. However, in other studies, no significant differences in the PUFA content were observed between the tested sausage samples after 21 days of fermentation [55]. These values were similar, which proves that acid whey protects polyunsaturated fatty acids against oxidative changes, as well as curing salt. Karwowska and Dolatowski [56] also found that the use of acid whey in uncured venison sausages did not significantly affect the PUFA content after production and was similar to the control sample. Karwowska and Kononiuk [20] also found that during a 21-day fermentation of beef sausages with acid whey, no statistically significant differences in the PUFA content were observed. The lack of these differences was explained by a similar level of the TBARS index throughout the fermentation period, which proves the high oxidative stability of acid whey sausages.

Based on the results of this study and those of other authors, it can be concluded that changes in the profile of fatty acids in meat products are influenced by both internal factors, i.e., the species, the share of adipose tissue, heme proteins, metals, pro-oxidative enzymes, vitamins, antioxidant enzymes, and peptides, and external factors, such as processing conditions (process parameters, grinding, and thermal treatment), additives (acid whey, nitrate and nitrite, sodium ascorbate, and plant extracts), and storage conditions [11,20,22,54,55].

In the present study, interesting results were obtained in terms of the fermented pork bacon fatty acid profile during storage. It was shown that the PUFA decreased and SFA increased significantly for the nitrite treatment (C). The treatment and time of storage affected (*p* < 0.001) the content of n-3 and n-6 fatty acids. An interaction between the treatment and time of storage for n-3 and n-6 also was found (Table 5). In the C treatment, it was shown that the content of n-3 and n-6 fatty acids also decreased after storage (*p* < 0.05). The PUFA content was lower than those obtained after fermentation (time 0), probably due to their high susceptibility to oxidation [49]. Other authors have also observed a decrease in PUFA levels during the refrigerated storage of fermented products, which was the reason for greater susceptibility to oxidation and hydrolysis [22,54,55]. The opposite tendency was observed in the acid whey treatment (AW), where the PUFA increased; likewise, the content of n-3 and n-6 fatty acids increased and SFA decreased significantly during storage (*p* < 0.05) (Table 5). After four weeks of storage, in the AW treatment, a significant increase (*p* < 0.05) in the content of PUFA (by 2.25%) was observed and recorded in comparison to the value, which was measured directly after ripening.

The acid whey used in this experiment contained a high level of lactic acid bacteria (approximately 8.0 log CFU/mL). Some studies have indicated that lactic acid bacteria lipases are involved in the lipid metabolism of fermented meat products [30]. The results of these studies suggest that LAB from acid whey could potentially be lipolytic active; this could explain the increased PUFA ratio in the meat. Similar observations concerning the influence of LAB on the fatty acid profile were made by Najjari et al. [57] in Tunisian dry-fermented sausages with *Lactobacillus sakei*, and by Arief et al. [58] in fermented beef sausage with *Lactobacillus plantarum* IIA-2C12 or *Lactobacillus acidophilus* IIA-2B4. Nevertheless, still, little information is available concerning lipases from lactic acid bacteria. Some published data suggest the role of fatty acids and fats as essential growth factors for LAB [59,60,61]. The standard MRS medium, which is used for the nonselective cultivation of lactobacilli, contains 0.1% (*w*/*v*) Tween 80, a water-soluble derivative of oleic acid (cis-9-octadecenoic acid), which is known to enhance the growth of many LAB [62]. Furthermore, most LAB are grown to incorporate oleic acid into their membranes and to further convert them into cyclopropane fatty acids, characteristic of fatty acids, especially in lactobacilli. The importance of cyclopropane fatty acids in LAB membranes increases the fluidity of the membrane like polyunsaturated fatty acids and protects LAB from adverse environmental effects such as extreme temperatures, low pH, deleterious effects of oxygen, and entering a stationary growth phase [63].

During storage, MUFA decreased in the C and AW treatments. However, in the T treatment, MUFA significantly increased and PUFA and SFA decreased during storage (*p* < 0.05). In the nitrate treatment, the highest number of lactic acid bacteria (7.18 log CFU/g) was found after storage (Table 5); thus, the influence of environmental bacteria on changes in the profile of fatty acids in the T treatment cannot be excluded.

The fatty acid profiles of the fermented bacon changed over time 0 (after fermentation) and after storage. In the T treatment, the lowest PUFA content (7.60%) was observed after storage. The lowest content of MUFA was observed in the C treatment (50.35%), while a higher content was found in the AW treatment (51.15%), and the highest was in the T treatment (56.80%) (*p* < 0.05). In the T treatment, the lowest SFA content was also found (*p* < 0.05). The SFA content in the C and AW treatments was at a similar level (Table 5).

The treatment affected (*p* < 0.001) the PUFA/SFA ratio, while the time of storage did not (*p* > 0.05). An interaction between the treatment and time of storage for the PUFA/SFA ratio was found (Table 5). The PUFA/SFA ratio is an indicator of the nutritional value of food, and higher values of this index are considered beneficial to human health [64]. In our study, the PUFA/SFA ratio after production ranged from 0.24 to 0.40, and after four weeks of refrigerated storage, it ranged from 0.21 to 0.35 and indicated a loss of polyunsaturated fatty acids in the nitrite and nitrate treatments. The exception was the AW treatment, where an increase in the index value was noted (by 0.08), and thus the nutritional value of the acid whey bacon improved. The decrease in the PUFA/SFA ratio after four weeks of refrigerated storage may be due to the loss of PUFA through hydrolysis and oxidation [49].

### 3.5. Sensory Analysis

**Figure 1:**
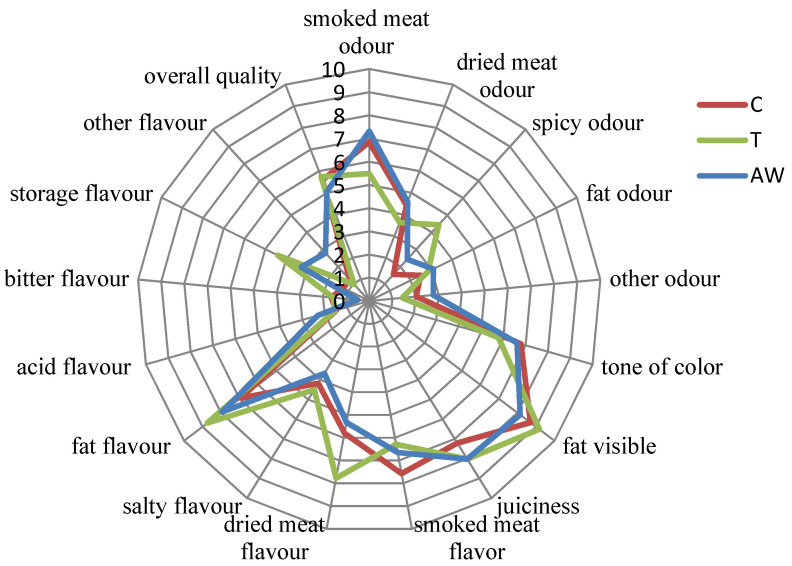
Sensory profile of the fermented pork bacon.

The results of the sensory evaluation of the experimental bacon after fermentation, as conducted by the QDP method, are presented in Figure 1. The statistical results of the sensory evaluation of the experimental bacon are summarized in the Supplementary Table S1. Flavor and odor are important attributes of food quality, determining greatly consumer preferences in this respect [41,65]. The evaluated products were characterized by the intensive odor and flavor of the smoked meat, with a weak intensity of feeling for the odor of dried meat, sharp odor, and other odors. In a cross-section of a bacon slice, the pink color of the meat and the bright white color of the fat were visible. In the examined treatment of fermented bacon, the intensity of the fat content was highly evaluated (ca. 8 c.u.); however, no significant differences between the particular samples were not found (*p* > 0.05). All treatments of the fermented bacon were characterized by a mean overall quality at the level of ca. 5.8 c.u.

The nitrite (C) and acid whey (AW) treatments were characterized by the highest intensity of smoked meat odor. In turn, the highest intensity of spice odor was shown in the T treatment (*p* < 0.05). The fermented bacon with the addition of acid whey (AW) and nitrate (T) revealed the highest intensity of fat odor. Simultaneously, a higher average note for storage flavor was found in the T and AW treatments than in the C treatment. Nevertheless, these factors did not significantly influence the overall quality evaluation of the products (*p* > 0.05). After production, a significantly lower polyunsaturated fatty acid content for the sample with nitrate and acid whey was also found (Table 5). Furthermore, the AW treatment was characterized by the highest TBARS index value (Table 4). The unpleasant flavor of rancid fat in food is a result of the release of low-molecular volatile compounds, i.e., short-chained aldehydes, or the resulting oxidation-induced acids [66,67]. It seems that the amount of compounds generated as a result of oxidative changes in the AW and T treatments was not high enough to cause any significant differences in the overall sensory quality of these compounds and thus were masked by other lactic acid, peptides, and free amino acids that could have affected the sensory perceptions of the evaluators [68]. Furthermore, oxidative changes in fermented/ripened products may positively affect the generation of appropriate flavoring. Products that are allowed to mature for several months have a stronger taste and aroma and a higher concentration of oxidation products responsible for the aroma and flavor of the product [69]. The meat tissue in all of the fermented bacon was reddish-pink in color and characteristic for nitrosylmyoglobin (MbFe^2+^NO) occurring in cured meat products [70]. The pink color of the meat tissue in the bacon with the addition of nitrite (C) and acid whey (AW) was evaluated as being darker. As suggested by other authors, in uncured fermented meat products with the addition of acid whey, nitrite oxide (NO) can be produced by the bacterial enzyme NO synthase (bNOS), which catalyzes the oxidation of L-arginine to L-citrulline. NO can react with myoglobin, forming MbFe^2+^NO [8]. The addition of acid whey to the fermented bacon lowered the intensity of the smoked meat flavor (*p* < 0.05).

The intensity of the dried meat flavor was the highest in the T treatment (*p* < 0.05). There were no statistically significant differences in the intensity of dried meat flavor between treatment C and AW (*p* > 0.05). The highest value of visible fat was shown in the variant T, and this result was probably related to the light pink color of the meat tissue found in this treatment (Figure 1). The products with nitrate (T) were evaluated as being juicier in respect to taste. This observation may be related to the acidity of the products. The T treatment was characterized by the highest pH (Table 2). It is known that a higher pH increases the juiciness of the meat [71].

## 4. Conclusions

Based on our studies, it was found that nitrite and nitrate treatments showed better lipid protection during bacon fermentation than acid whey. This is indicated by the fatty acid profile of fermented pork bacon and the value of the TBARS index after production. On the contrary, acid whey positively changed the sensorial characteristics of uncured fermented pork bacon directly after the ripening process and had the effect of a decrease in the pH of the product. Uncured fermented pork bacon with acid whey was characterized by an overall quality similar to cured treatment, which could be an important factor for consumers. Moreover, acid whey, in contrast to nitrite treatment, positively changed the fatty acid profile during storage, where PUFA increased; likewise, the content of n-3 and n-6 fatty acids increased and SFA decreased. After storage, the nutritional value of the fermented bacon with acid whey improved due to the PUFA/SFA ratio increased. After storage, the amount of secondary oxidation products (TBARS) was similar in the variant with acid whey and in the variant with nitrite, but a significantly higher TBARS index value was found in the nitrate treatment.

The mechanism of the effect of acid whey on oxidation changes in meat products with a high fat content is not currently fully understood. An interesting direction that should be investigated is the influence and mechanism of the action of lactic acid bacteria that come from acid whey on the lipid metabolism in fermented meat products. Further studies on the evaluation of the effect of acid whey on sensory quality, oxidative processes, shelf-life, and microbiology during the long time storage of traditional Polish fermented bacon are necessary.

## Figures and Tables

**Table 1:** Definition of the sensory attributes.

Discriminants	Definition	Boundary Terms
Flavor		
Smoked meat	Intensity flavor of volatile organic compounds from the thermal decomposition of wood	0 = not intensive–10 = very intensive
Dried meat	Characteristic of dry fermented meat	0 = not intensive–10 = very intensive
Salty	Intensity of salty flavor	0 = not intensive–10 = very intensive
Fatty	Intensity of a fatty flavor	0 = not intensive–10 = very intensive
Acid	Intensity of an acid flavor	0 = not intensive–10 = very intensive
Bitter	Intensity of a bitter flavor	0 = not intensive–10 = very intensive
Stored	Lack of freshness	0 = not intensive–10 = very intensive
Other	Other sensations not on the list	0 = not intensive–10 = very intensive
Odor		
Smoked meat	Intensity of the smoked odor	0 = not intensive–10 = very intensive
Dried meat	Intensity of a dried meat odor	0 = not intensive–10 = very intensive
Spicy	Intensity of an irritating impression when smelling	0 = not intensive–10 = very intensive
Fatty	Intensity of a fatty odor	0 = not intensive–10 = very intensive
Other	Other sensations, not on the list	0 = not intensive–10 = very intensive
Appearance		
Tone of color	Intensity of the red color associated to meat tissue (cured meat color)	0 = light pink–10 = dark pink
Fat visible	The amount of fat visible in a cross-section of the product	0 = low amount–10 = high amount
Juiciness	The appearance of juiciness in the product, which is a combination of fat and water	0 = not very juicy–10 = very juicy
Overall quality	Attribute of the total quality of dry fermented pork bacon	0 = low–10 = very high

**Table 2:** Microbiological assessment of fermented pork bacon (means ± standard deviations).

	Treatment	Number of Microorganisms (log CFU/g)		Treatment	Time of Storage	Treatment × Time of Storage
Storage time (weeks)		0	4	*p*	*p*	*p*
TVC	C	6.40 ± 0.17 ^aD^	6.36 ± 0.21 ^bD^	***	***	***
	T	6.48 ± 0.22 ^aD^	7.32 ± 0.14 ^cE^			
	AW	6.23 ± 0.25 ^aD^	6.70 ± 0.09 ^aE^			
LAB	C	5.34 ± 0.26 ^aD^	6.08 ± 0.15 ^aE^	***	***	*
	T	6.00 ± 0.41 ^bD^	7.18 ± 0.08 ^bE^			
	AW	6.72 ± 0.17 ^cD^	6.90 ± 0.23 ^cD^			
ENT	C	<1.00	<1.00	***	***	***
	T	<1.00	<1.00			
	AW	<1.00	2.78 ± 0.32 ^aD^			
Y&M	C	6.54 ± 0.21 ^bE^	5.30 ± 0.43 ^aD^	*	***	*
	T	5.70 ± 0.09 ^aD^	5.60 ± 0.41 ^aD^			
	AW	6.58 ± 0.18 ^bE^	5.90 ± 0.24 ^aD^			
STA	C	<1.00	<1.00	NS	NS	NS
	T	<1.00	<1.00			
	AW	<1.00	<1.00			
LIST	C	nd	nd	NS	NS	NS
	T	nd	nd			
	AW	nd	nd			

^a–c^ Means followed by different lowercase letters between the treatment at the same storage time and ^D,E^ capital letters between the same treatment at different storage times are significantly different (*p* < 0.05); *p*, significance of effects, treatment, time of storage, and treatment × time of storage interaction; NS, not significant; * *p* < 0.05; *** *p* < 0.001; C, control bacon with a nitrite curing mixture (99.5% NaCl, 0.5% NaNO_2_); T, bacon with a nitrate curing mixture (99.5% NaCl, 0.5% NaNO_3_); AW, bacon with acid whey and NaCl; TVC, total viable count; LAB, lactic acid bacteria; ENT, *Enterobacteriaceae*; Y&M, yeast and mold; STA, *Staphylococcus aureus*; LIST, *Listeria monocytogenes*; nd, not detected.

**Table 3:** Values of pH and potential redox (ORP) of fermented pork bacon (means ± standard deviations).

	Treatment	Storage Time (Weeks)		Treatment	Time of Storage	Treatment × Time of Storage
		0	4	*p*	*p*	*p*
pH	C	5.89 ± 0.01 ^bD^	5.90 ± 0.01 ^bD^	***	***	***
	T	6.06 ± 0.01 ^cE^	6.00 ± 0.04 ^cD^			
	AW	5.78 ± 0.01 ^aE^	5.67 ± 0.01 ^aD^			
ORP (mV)	C	282.60 ± 1.87 ^aD^	300.50 ± 1.65 ^aE^	***	***	***
	T	287.87 ± 1.42 ^bD^	304.33 ± 0.68 ^bE^			
	AW	363.13 ± 1.57 ^cE^	347.00 ± 3.32 ^cD^			

^a–c^ Means followed by different lowercase letters between the treatment at the same storage time and ^D,E^ capital letters between the same treatment at different storage times are significantly different (*p* < 0.05); *p*, significance of effects, treatment, time of storage, treatment × time of storage interaction; *** *p* < 0.001; C, control bacon with a nitrite curing mixture (99.5% NaCl, 0.5% NaNO_2_); T, bacon with a nitrate curing mixture (99.5% NaCl, 0.5% NaNO_3_); AW, bacon with acid whey and NaCl.

**Table 4:** TBARS index value of fermented pork bacon (means ± standard deviations).

	Treatment	Storage Time (Weeks)		Treatment	Time of Storage	Treatment × Time of Storage
		0	4	*p*	*p*	*p*
TBARS (mg/kg)	C	0.96 ± 0.03 ^bE^	0.70 ± 0.03 ^aD^	***	***	***
	T	0.71 ± 0.05 ^aD^	1.62 ± 0.03 ^bE^			
	AW	1.68 ± 0.05 ^cE^	0.73 ± 0.02 ^aD^			

^a–c^ Means followed by different lowercase letters between the treatment at the same storage time and ^D,E^ capital letters between the same treatment at different storage times are significantly different (*p* < 0.05); *p*, significance of effects, treatment, time of storage, and treatment × time of storage interaction; *** *p* < 0.001; C, control bacon with a nitrite curing mixture (99.5% NaCl, 0.5% NaNO_2_); T, bacon with a nitrate curing mixture (99.5% NaCl, 0.5% NaNO_3_); AW, bacon with acid whey and NaCl.

**Table 5:** Fatty acid composition (% total fatty acids) in experimental pork bacon after fermentation and four weeks of cold storage.

	Treatment	Storage Time (Weeks)		Treatment	Time of Storage	Treatment × Time of Storage
		0	4	*p*	*p*	*p*
Ʃ SFA	C	34.77 ± 0.02 ^aD^	36.80 ± 0.04 ^bE^	***	***	***
	T	39.02 ± 0.22 ^bE^	35.55 ± 0.03 ^aD^			
	AW	39.00 ± 0.01 ^bE^	36.70 ± 0.07 ^bD^			
Ʃ MUFA	C	51.37 ± 0.02 ^abE^	50.35 ± 0.09 ^aD^	***	***	***
	T	50.90 ± 0.10 ^aD^	56.80 ± 0.07 ^cE^			
	AW	51.55 ± 0.03 ^bE^	51.15 ± 0.05 ^bD^			
Ʃ PUFA	C	13.75 ± 0.01 ^Be^	12.70 ± 0.03 ^cD^	***	***	***
	T	9.75 ± 0.02 ^aE^	7.60 ± 0.00 ^aD^			
	AW	9.55 ± 0.01 ^aD^	11.80 ± 0.02 ^bE^			
n-3	C	1.30 ± 0.01 ^cE^	1.20 ± 0.02 ^cD^	***	***	***
	T	0.83 ± 0.02 ^aE^	0.60 ± 0.03 ^aD^			
	AW	0.90 ± 0.02 ^bD^	1.10 ± 0.01 ^bE^			
n-6	C	11.75 ± 0.05 ^cE^	10.90 ± 0.02 ^cD^	***	***	***
	T	8.38 ± 0.15 ^bE^	6.40 ± 0.05 ^aD^			
	AW	8.05 ± 0.05 ^aD^	10.20 ± 0.03 ^bE^			
Ʃ PUFA/Ʃ SFA	C	0.40 ± 0.02 ^bE^	0.35 ± 0.02 ^bD^	***	NS	***
	T	0.25 ± 0.01 ^aE^	0.21 ± 0.01 ^aD^			
	AW	0.24 ± 0.04 ^aD^	0.32 ± 0.03 ^bE^			

^a–c^ Means followed by different lowercase letters between the treatment at the same storage time and ^D,E^ capital letters between the same treatment at different storage times are significantly different (*p* < 0.05); *p*, significance of effects, treatment, time of storage, and treatment × time of storage interaction; NS, not significant; *** *p* < 0.001; C, control bacon with a nitrite curing mixture (99.5% NaCl, 0.5% NaNO_2_); T, bacon with a nitrate curing mixture (99.5% NaCl, 0.5% NaNO_3_); AW, bacon with acid whey and NaCl; SFA, saturated fatty acid; MUFA, monounsaturated fatty acid; PUFA, polyunsaturated fatty acid; n-3, omega-3; n-6, omega-6.

## Data Availability

The data presented in this study are available in article.

## Supplementary Materials

The following are available online at https://www.mdpi.com/article/10.3390/antiox10111711/s1, Table S1: Sensory quality discriminants of experimental bacon after fermentation (means ± standard deviations); Figure S1: Sample chromatogram of the fatty acids of fermented pork bacon with the use of acid whey (AW) after 4 weeks of storage.
