# Supplementary material for: The Influence of Acid Whey on the Lipid Composition and Oxidative Stability of Organic Uncured Fermented Bacon after Production and during Chilling Storage
Source: Antioxidants (Basel). 2021 Oct 27;10(11):1711. doi: 10.3390/antiox10111711 (PMC8614668; doi:10.3390/antiox10111711)
Supplement: Supplementary file 1 [file antioxidants-10-01711-s001.zip › antioxidants-1402597-supplementary.pdf]

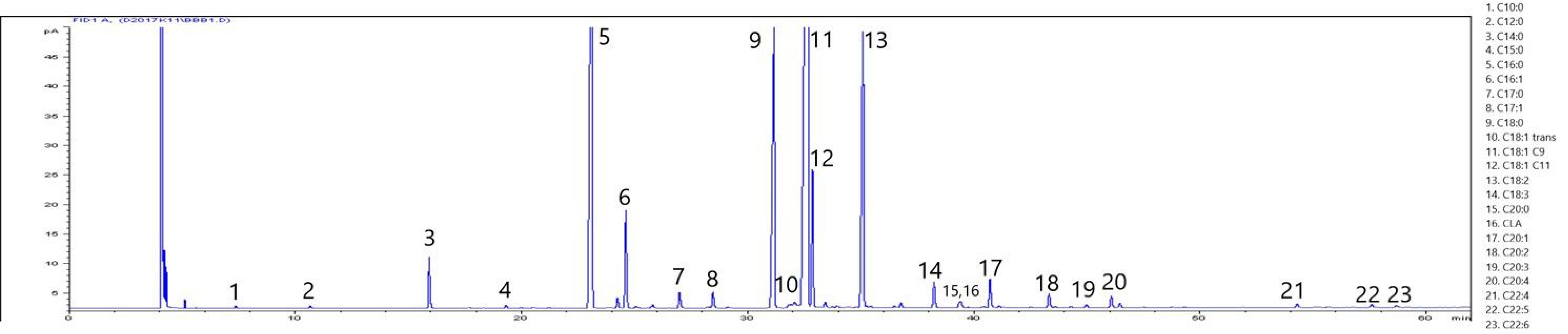

Figure S1. Sample chromatogram of fatty acids of the fermented pork bacon with the use of acid whey (AW) after 4 weeks of storage.

Table S1. Sensory quality discriminants of experimental bacons after fermentation (means ± standard deviation).

Sensory quality features	Treatment	Intensity [0-10 c.u]
Smoked meat flavour	C	7.58±0.59[b]
	T	6.29±1.02[a]
	AW	6.65±1.14[b]
Dried meat flavour	C	5.80±1.45[a]
	T	7.77±1.24[c]
	AW	5.33±1.25[b]
Salty flavour	C	4.18±1.58[a]
	T	4.50±2.93[a]
	AW	3.67±1.44[a]
Fatty flavour	C	6.96±1.44[a]
	T	8.73±0.90[b]
	AW	7.94±1.33[b]
Acid flavour	C	1.49±1.42[a]
	T	1.53±1.15[a]
	AW	2.31±1.46[a]
Bitter flavour	C	1.65±1.97[a]
	T	1.56±2.53[a]
	AW	0.51±0.57[a]
Storage flavour	C	1.25±1.39[b]
	T	4.41±1.10[a]
	AW	3.27±1.81[a]
Other flavour	C	1.24±1.98[a]
	T	0.97±2.02[a]
	AW	2.79±2.42[a]
Smoked meat odour	C	6.85±0.80[a]
	T	5.49±1.62[b]
	AW	7.31±0.90[a]
Dried meat odour	C	4.43±1.27[a]
	T	3.62±1.86[a]
	AW	4.57±1.13[a]

Spicy odour	C	1.55±1.45[a]
	T	4.45±2.01[b]
	AW	2.96±1.58[ab]
Fatty odour	C	2.48±1.73[a]
	T	2.82±1.85[a]
	AW	3.09±0.95[a]
Other odour	C	2.03±1.93[a]
	T	1.43±1.92[a]
	AW	2.77±2.28[a]
Ton of colour	C	6.80±1.32[b]
	T	5.79±1.37[a]
	AW	6.62±1.15[b]
Fat visible	C	8.70±1.07[ab]
	T	9.18±0.90[b]
	AW	8.15±1.07[a]
Juiciness	C	7.21±1.28[ab]
	T	8.02±1.17[b]
	AW	6.85±1.49[a]
Overall quality	C	5.59±1.17[a]
	T	5.75±0.99[a]
	AW	5.06±1.01[a]

[a-c] Means followed by the different lower case letters between the treatments at the same storage time ($P < 0.05$); C - control bacon with the nitrite curing mixture (99.5% NaCl, 0.5% $NaNO_2$); T- bacon with the nitrate curing mixture (99.5% NaCl, 0.5% $NaNO_3$) and AW- bacon with acid whey and NaCl;
